# Event and Comment

**Published:** 1923-07

**Authors:** 


### DENTAL REGISTER

THE MAGAZINE OF DENTISTRY

Vol. LXXVII JULY, 1923 No. 7

### EVENT AND COMMENT

BY VOTRE AMI DENTAIRE

You can not have temperance if you have prohibition. Men and women, whom, by our adoration today we make immortal, had a hard struggle for existence when they were alive—why be hard on the present generation. It is the presumption that something will be put over on you that such is done.

A man's ability to stay in one place a life-time is his willingness to be true to those to whom he originally signified allegiance. Those to whom you give your most loyal support often fail to recognize your fealty because they do not realize that their eminence depends upon your support.

Correlations of the mind which come from certain kaleidoscopic relations of ideas often produce facts that lead to inventions undreampt of—there is much yet for the human mind to discover.

A friend in need is a friend indeed, for a friend in deed is rare. If a friend you need comes with adequate speed he is above all more than fair.

The very people who say you are a fool and a crazy idiot are the very ones who afterward have to admit that you are a wise man—having wisdom abounding.

It is not the drink of the devil, it is the nectar of the gods.

Bordeaux Madam? The boat steward says this so many times during the day that he says it in his sleep also. Why are we robbed of a gentleman drink? Russia without vodka is like United States without rye. Vodka is the essential active principle spirit of rye.

